# Self-esteem as a mediator between perfectionism and mental health outcomes

**DOI:** 10.1080/00049530.2026.2613533

**Published:** 2026-01-18

**Authors:** Maya Curtis, David Garratt-Reed

**Affiliations:** Psychology, Curtin University, Perth, Western Australia, Australia

**Keywords:** Perfectionistic concerns, perfectionistic strivings, self-esteem, psychological distress, flourishing

## Abstract

**Objective:**

In line with the Two Continua Model of Mental Health, this study investigated whether self-esteem (SE) mediates the relationship between two dimensions of perfectionism (perfectionistic concerns (PC) and perfectionistic strivings (PS)) and negative and positive mental health outcomes, including psychological distress (PD) and flourishing (F).

**Method:**

Participants (*N* = 162; 121 females, 36 males, 4 non-binary, and 1 not disclosed) aged 18 to 90 (*M* = 31.57, SD = 15.75) completed an online survey containing five measures.

**Results:**

There was a significant positive indirect effect between PC and PD through SE (b = 0.39, *p* < .001, 95% CI [0.27, 0.51]) and between PS and F through SE (b = 0.14, *p* = .004, 95% CI [0.05, 0.24]). There was a significant negative indirect effect between PC and F through SE (b = −0.48, *p* < .001, 95% CI [−0.62, −0.35]) and between PS and PD through SE (b = -0.12, *p* = .004, 95% CI [−0.20, −0.04]).

**Conclusions:**

Findings support the utility of the Two Continua Model, contribute to literature on the relationship between perfectionism and mental health outcomes, and highlight the potential utility of exploring therapeutic interventions to target self-esteem for individuals high in perfectionism.

## KEY POINTS

**What is already known about this topic:**

1. PC has been consistently linked to negative mental health outcomes.
2. PS has been inconsistently linked with negative and positive mental health outcomes.
3. Self-esteem mediates the relationship between variables that are similar to perfectionism and negative mental health outcomes.

**What this topic adds:**

1. Self-esteem mediated the relationship between higher PC and higher psychological distress and lower flourishing.
2. Self-esteem mediated the relationship between higher PS and lower psychological distress and higher flourishing.
3. Lower or higher self-esteem may be a risk or protective factor, respectively, in the relationship between perfectionism and mental health outcomes.

## Introduction

Perfectionism is a multidimensional construct that refers to an individual’s pursuit of exceedingly high standards and self-criticism for not meeting such standards (Frost et al., 1990; Hewitt & Flett, 1991). There are several approaches to measuring perfectionism, such as Hewitt and Flett’s (1991) Multidimensional Perfectionism Scale which measures three dimensions of perfectionism, including self-oriented perfectionism, other-oriented perfectionism, and socially prescribed perfectionism. However, more recent research by Burgess et al. (2016) now supports measuring two dimensions of perfectionism, namely perfectionistic concerns and perfectionistic strivings.

Perfectionistic concerns refer to self-criticism for not achieving highly set goals and concern about negative performance evaluation (Burgess et al., 2016). Perfectionistic strivings refers to setting exceedingly high goals and striving for perfection in achieving goals (Burgess et al., 2016). Limburg et al. (2017) outline the constructs that are classified under perfectionistic concerns, including socially prescribed perfectionism, self-critical perfectionism, negative perfectionism, discrepancy, concern over mistakes, doubts about actions, parental expectations, and parental criticism. They also explain that perfectionistic strivings include self-oriented perfectionism, other-oriented perfectionism, personal and high standards, and organisation. The terms “perfectionistic concerns” and “perfectionistic strivings” will be used throughout this article to encapsulate the constructs that fall under each of them.

Perfectionism is a well-established predictor of negative mental health outcomes, such as psychological distress, and further research has explored mediating factors in this relationship, with a focus on self-esteem (e.g., Chai et al., 2020; Rice et al., 1998). Rice et al. (1998) found that self-esteem mediated the effect of perfectionistic concerns on depression, yet perfectionistic strivings was not associated with depression directly or through self-esteem. However, the relationship between perfectionism and positive mental health outcomes, both directly and through self-esteem, has been less extensively studied. The need to examine this relationship is underscored by the Two Continua Model of Mental Health, which proposes that psychological distress and flourishing are two related yet distinct continuums, such that the absence of one is not synonymous with the presence of the other (Keyes, 2002; Westerhof & Keyes, 2010). Psychological distress refers to symptoms of depression, anxiety, and stress (Lovibond & Lovibond, 1995), and flourishing refers to high positive wellbeing and optimal functioning in different psychosocial domains (Butler & Kern, 2016). This gap highlights the need for further investigation into the relationship between perfectionism and flourishing through self-esteem, to contribute to a more holistic understanding of the link between perfectionism and mental health.

### Perfectionism and mental health outcomes

Multiple studies support the relationship between perfectionism and negative mental health outcomes. Geranmayepour and Besharat (2010) found that higher perfectionistic concerns were correlated with higher psychological distress and lower psychological wellbeing (similar to flourishing) among 185 university students. This is supported by similar findings that higher perfectionistic concerns predicted higher psychological distress (Eley et al., 2020; Kahn et al., 2022; Wimberley & Stasio, 2013) and was correlated with lower flourishing (Flett & Hewitt, 2015; Stoeber & Corr, 2016).

Recent systematic reviews and meta-analyses found that higher perfectionistic concerns had moderate positive correlations with symptoms of anxiety, obsessive-compulsive disorder, and depression among children, adolescents, and adults, whereas perfectionistic strivings had small positive correlations with these outcomes (Callaghan et al., 2023; Lunn et al., 2023). These findings suggest that perfectionistic concerns are more strongly related to negative mental health outcomes than perfectionistic strivings.

Some studies suggest a positive relationship between perfectionistic strivings and positive mental health outcomes. Perfectionistic strivings correlated with lower psychological distress and higher psychological wellbeing and flourishing in university student samples (Geranmayepour & Besharat, 2010; Stoeber & Corr, 2016). This is in line with Ståhlberg et al. (2019) finding that the combination of high perfectionistic strivings and low perfectionistic concerns was positively related to mastery goals, motivation, and wellbeing among university students. Thus, the relationship between higher perfectionistic strivings and higher positive mental health outcomes may be common in university students. However, these findings are at odds with the wider literature conducted across different samples (e.g., Callaghan et al., 2023; Lunn et al., 2023) in which perfectionistic strivings are more frequently found to be positively related to negative mental health outcomes.

There is a well-established relationship between higher perfectionistic concerns and higher psychological distress (Eley et al., 2020; Geranmayepour & Besharat, 2010; Kahn et al., 2022; Wimberley & Stasio, 2013), and some research supports the relationship between higher perfectionistic concerns and lower flourishing (e.g., Stoeber & Corr, 2016). Research also highlights the link between perfectionistic strivings and psychological distress and flourishing. It is important to explore the processes by which perfectionistic concerns and perfectionistic strivings relate to mental health outcomes through possible mediators, such as self-esteem.

### Self-esteem and mental health outcomes

Self-esteem is an individual’s evaluations about their own importance and self-worth (Moksnes & Reidunsdatter, 2019). Lower self-esteem predicts more negative mental health outcomes, including psychological distress, depression, and anxiety (Doyle & Catling, 2022; Li et al., 2010). Higher self-esteem predicts more positive mental health outcomes, including life satisfaction and mental wellbeing (similar to flourishing; Lee, 2020; Trong Dam et al., 2023). As demonstrated so far, perfectionism and self-esteem both predict mental health outcomes, yet evidence also indicates that perfectionism predicts self-esteem.

### Perfectionism and self-esteem

Several studies support the relationship between perfectionism and self-esteem. Ashby and Rice (2002) found that perfectionistic concerns and perfectionistic strivings were negative and positive predictors of self-esteem, respectively. More recent studies have replicated and extended these findings. Perfectionistic concerns consistently show moderate to strong negative correlations with self-esteem (e.g., Dunkley et al., 2012; Juwono et al., 2022; Taylor et al., 2016). A meta-analysis of the effects of perfectionistic concerns on self-esteem confirms this with a pooled association of *r* = −.42 (Khossousi et al., 2024). However, perfectionistic strivings show mixed findings of small negative (Dunkley et al., 2012) and positive (Khossousi et al., 2024; Taylor et al., 2016) correlations with self-esteem. This research suggests that higher perfectionistic concerns are more strongly related to lower self-esteem whereas higher perfectionistic strivings are weakly and inconsistently related to self-esteem. These findings suggest the utility of exploring self-esteem as a potential mediating factor in the relationship between perfectionism and mental health outcomes more broadly.

### Perfectionism, self-esteem, and mental health outcomes

Little is known about the mediating role of self-esteem in the well-established relationships between perfectionism and both negative and positive mental health outcomes. Most research on the relationship between perfectionism and mental health outcomes has assessed elements of, rather than overall, psychological distress and has not directly assessed flourishing. This highlights the need to investigate these relationships using overall measures of psychological distress and flourishing, in line with the Two Continua Model of Mental Health.

Studies have found that higher perfectionistic concerns were indirectly related to higher negative mental health outcomes, including depression and anxiety, through self-esteem (Ashby et al., 2006; Chai et al., 2020; Kempke et al., 2011; Lasota & Kearney, 2017). Chai et al. (2020) also found that higher perfectionistic strivings were indirectly related to lower depression through self-esteem. However, these studies did not assess an overall measure of distress or any positive mental health outcomes.

Overall, the research discussed throughout this introduction presents mixed findings on the relationship between perfectionism, self-esteem, and mental health outcomes. This highlights the complex nature of perfectionism, particularly perfectionistic strivings. No study has examined whether perfectionistic concerns and perfectionistic strivings are indirectly related to psychological distress and flourishing, through self-esteem.

### The present study

This study investigates the relationship between perfectionistic concerns and perfectionistic strivings and psychological distress and flourishing through self-esteem as a mediator. It aims to replicate findings on the relationship between perfectionism and psychological distress through self-esteem (e.g., Ashby et al., 2006; Chai et al., 2020; Lasota & Kearney, 2017; Rice et al., 1998) using an overall measure of distress rather than a domain-specific (e.g., depression) measure and address the gap in the literature by examining the relationship between perfectionism and flourishing through self-esteem, in line with the Two Continua Model of Mental Health. Given that these earlier mediation studies were conducted decades ago, it is important to conduct this study to assess whether findings similar to those reported by Rice et al. (1998) and Ashby et al. (2006) can be replicated in the current context and whether there are changes in patterns when looking at perfectionism and both negative and positive mental health outcomes. This study also seeks to contribute to the growing evidence of mediating factors between perfectionism and mental health outcomes.

It is hypothesised that there will be a statistically significant positive indirect effect between perfectionistic concerns and psychological distress (H1) and a statistically significant negative indirect effect between perfectionistic concerns and flourishing (H2) through self-esteem. It is also hypothesised that there will be a statistically significant positive indirect effect between perfectionistic strivings and psychological distress (H3) and a statistically significant negative indirect effect between perfectionistic strivings and flourishing (H4) through self-esteem.

## Method

### Research design

A correlational cross-sectional research design was used. The hypotheses were tested through the Mediation Analysis option in Jeffrey’s Amazing Statistics Program (JASP) version 0.18 (JASP Team [JASP], 2023).

### Participants

The population of interest was all individuals aged 17 and older. Convenience and snowball sampling were used to recruit a sample from the general community and the university research participant pool of undergraduate psychology students. All students recruited via the participant pool received research points for their participation whereas participants from the general community did not receive anything. The required sample size with a power set at .80 for mediation with bias-corrected bootstrapping was 148 (Fritz & MacKinnon, 2007). This sample size was calculated using halfway to medium effect sizes for the “a” (effect between perfectionistic concerns or perfectionistic strivings and self-esteem) and “b” (effect between self-esteem and psychological distress or flourishing) pathways to ensure that the study was not underpowered. However, previous research found stronger relationships between these variables (Awad et al., 2022; Kempke et al., 2011; Miegel et al., 2020). After data screening, the final sample consisted of 162 participants (121 females, 36 males, 4 non-binary, and 1 not disclosed) and age ranged from 18 to 90 (*M* = 31.57, *SD* = 15.75).

### Measures

#### Demographics

Participants provided demographic data, including their age and gender. The response options for gender included man/male, woman/female, non-binary (please specify), or I prefer not to answer.

#### Frost Multidimensional Perfectionism Scale-Brief

The Frost Multidimensional Perfectionism Scale-Brief (F-MPS-Brief; Burgess et al., 2016) is an eight-item self-report questionnaire consisting of two subscales: perfectionistic concerns (four items) and perfectionistic strivings (four items). It uses a five-point Likert scale from one (strongly disagree) to five (strongly agree) for a minimum subscale score of four to a maximum subscale score of 20. Higher scores indicate more perfectionistic tendencies. The F-MPS-Brief has good internal consistency for perfectionistic concerns (α = .85 and .83) and perfectionistic strivings (α = .85 and .81) factors in clinical and community samples (Burgess et al., 2016). The present study also found good internal consistency for perfectionistic concerns (α = .87) and perfectionistic strivings (α = .85). The F-MPS-Brief has good construct validity as perfectionistic concerns are strongly correlated with the Almost Perfect Scale-Revised (APS-R) discrepancy subscale and perfectionistic strivings strongly correlated with the APS-R standards subscale (Burgess et al., 2016). It also has good convergent validity as perfectionistic concerns correlated with measures of general anxiety, depression, fear of negative evaluation, and distress (Burgess et al., 2016; Woodfin et al., 2020).

#### Rosenberg Self-Esteem Scale

The Rosenberg Self-Esteem Scale (RSES; Rosenberg, 1965) is a 10-item self-report questionnaire that measures global self-esteem by measuring positive (five items) and negative (five items) feelings about the self. The negative feelings items are reverse-scored and all item response scores are added to compute the total score. The RSES uses a four-point Likert scale from one (strongly disagree) to four (strongly agree) for a minimum total score of 10 to a maximum total score of 40 whereby higher scores indicate higher self-esteem. The RSES has excellent internal consistency in adults in the United States (α = .91; Sinclair et al., 2010) and good convergent validity with academic self-efficacy (*r* = .44; Afari et al., 2012). The present study found good internal consistency for the RSES (α = .89).

#### The Depression Anxiety Stress Scales 21

The Depression Anxiety Stress Scales 21 (DASS-21; Lovibond & Lovibond, 1995) is a 21-item self-report questionnaire that comprises three subscales (depression, anxiety, and stress) which consist of seven items each to measure symptoms of depression, anxiety, and stress. It uses a four-point Likert scale to capture responses about symptoms experienced over the past week, and the total score of the combined subscale scores measures overall psychological distress, ranging from 0 to 63 with higher scores indicating higher psychological distress. In this study, psychological distress is conceptualised according to Lovibond’s and Lovibond (1995) definition as a combination of depression, anxiety, and stress symptoms. Therefore, the DASS-21 overall total score is used to measure psychological distress. The DASS-21 overall total score has excellent internal consistency in an Australian general adult sample (α = .94; Crawford et al., 2011) and in the present study (α = .94), as well as good convergent validity as it highly correlated with the Mood and Anxiety Symptom Questionnaire-90 in a sample of university students in the United States (*r* = .73; Osman et al., 2012).

#### PERMA-Profiler

The PERMA-Profiler (Butler & Kern, 2016) is a 16-item self-report questionnaire that measures flourishing across five domains of wellbeing, including positive emotion, engagement, relationships, meaning, and accomplishment (Seligman, 2011). It has three questions to assess each of the five domains and one question to assess happiness. It uses an 11-point Likert scale from zero (never) to 10 (always). The overall total flourishing score is determined by adding each item score and then dividing the total score by 16 to compute an average score ranging between zero to 10. Higher scores indicate higher flourishing. The current study used the overall PERMA-Profiler score to measure flourishing as research supports that the five domains of wellbeing load onto a single higher-order factor of general flourishing (Bartholomaeus et al., 2020). The PERMA-Profiler overall total score has excellent internal consistency in Australian adults (α = .93; Ryan et al., 2019) and in the present study (α = .94). The PERMA-Profiler overall total score also has good convergent validity as it highly correlated with similar measures of wellbeing, such as the Flourishing Scale (*r* = .84; Butler & Kern, 2016).

### Procedure

Prior to recruiting participants, ethics approval was obtained from Curtin University’s Human Research Ethics Committee (HRE2023-0292). Two online survey links containing information sheets, demographic items, the four measures, and debriefing sheets were developed using Qualtrics. One survey link was advertised on social media (Facebook and Instagram) to recruit adults from the general community and the other survey link was advertised on the university research participation system to recruit undergraduate psychology students. Participants were presented with an online information sheet outlining the study’s aims and potential risks, and provided their informed consent by clicking a checkbox before completing the survey.

Once all of the data was collected, the survey closed and the data was downloaded from Qualtrics and uploaded to JASP for data analysis.

## Results

### Data screening and missing values analysis

The data was uploaded to JASP. From the initial sample of 169 participants, seven cases were deleted due to having at least one entire scale of missing data. There was no further missing data and the final sample comprised 162 participants (121 females, 36 males, 4 non-binary, and 1 not disclosed). The descriptive statistics for each variable are reported in Table A1 in Appendix A. These statistics are similar to the means found in other studies (Doyle & Catling, 2022; Ryan et al., 2019; Sinclair et al., 2012). Bivariate correlations revealed that all predictor and outcome variables were correlated in the expected direction and magnitude (see Table B1 in Appendix B).

### Assumption testing

Assumptions of multiple regression analysis were checked and found to be satisfied before regression analyses were conducted.

### Data analysis

Regressions were modelled simultaneously via the Mediation Analysis option in JASP with 95% confidence intervals using 5000 bootstrap samples. Age was controlled for as it statistically significantly correlated with both outcome variables whereas gender was not controlled for as it did not statistically significantly correlate with both outcome variables. The regressions included perfectionistic concerns or perfectionistic strivings as the predictor, self-esteem as the mediator, and psychological distress or flourishing as the criterion variable (see Table 1).

**Table 1.** Total, direct, and indirect effects of each hypothesised pathway.

					95% Confidence Interval	
Total Effects	Estimate	Std. Error	z-value	*p*	Lower	Upper
PC → PD	0.47	0.07	6.38	<.001***	0.33	0.62
PC → F	−0.33	0.08	−3.94	<.001***	−0.49	−0.16
PS → PD	0.03	0.07	0.45	0.654	−0.10	0.16
PS → F	0.16	0.07	2.14	0.033*	0.01	0.30
Direct Effects						
PC → PD	0.08	0.08	1.09	0.275	−0.07	0.23
PC → F	0.16	0.08	2.02	0.044*	0.00	0.31
PS → PD	0.15	0.06	2.62	0.009**	0.04	0.25
PS → F	0.02	0.06	0.25	0.800	−0.10	0.13
Indirect Effects						
PC → SE → PD (H1)	0.39	0.06	6.42	<.001***	0.27	0.51
PC → SE → F (H2)	−0.48	0.07	−6.92	<.001***	−0.62	−0.35
PS → SE → PD (H3)	−0.12	0.04	−2.87	0.004**	−0.20	−0.04
PS → SE → F (H4)	0.14	0.05	2.91	0.004**	0.05	0.24

*Note*. PC = perfectionistic concerns, PS = perfectionistic strivings, PD = psychological distress, F = flourishing, SE = self-esteem, *p < .05., **p < .01., ***p < .001.

The total effect between perfectionistic concerns and psychological distress was statistically significant and positive (*b* = 0.47, *SE* = 0.07, *z* = 6.38, *p* < .001, 95% CI [0.33, 0.62]). The direct effect between perfectionistic concerns and psychological distress was positive and not statistically significant (*b* = 0.08, *SE* = 0.08, *z* = 1.09, *p* = .275, 95% CI [−0.07, 0.23]). There was a statistically significant positive indirect effect between perfectionistic concerns and psychological distress through self-esteem, supporting H1 (*b* = 0.39, *SE* = 0.06, *z* = 6.42, *p* < .001, 95% CI [0.27, 0.51]).

The total effect between perfectionistic concerns and flourishing was statistically significant and negative (*b* = −0.33, *SE* = 0.08, *z* = −3.94, *p* < .001, 95% CI [−0.49, −0.16]). Interestingly, the direct effect between perfectionistic concerns and flourishing was statistically significant and positive (*b* = 0.16, *SE* = 0.08, *z* = 2.02, *p* = .044, 95% CI [0.00, 0.31]). This direct effect was in the opposite direction to the total effect. There was a statistically significant negative indirect effect between perfectionistic concerns and flourishing through self-esteem, supporting H2 (*b* = −0.48, *SE* = 0.07, *z* = −6.92, *p* < .001, 95% CI [−0.62, −0.35]).

The total effect between perfectionistic strivings and psychological distress was not statistically significant (*b* = 0.03, *SE* = 0.07, *z* = 0.45, *p* = .654, 95% CI [−0.10, 0.16]). The direct effect between perfectionistic strivings and psychological distress was statistically significant and positive (*b* = 0.15, *SE* = 0.06, *z* = 2.62, *p* = .009, 95% CI [0.04, 0.25]). There was a statistically significant negative indirect effect between perfectionistic strivings and psychological distress through self-esteem, not supporting H3 (*b* = −0.12, *SE* = 0.04, *z* = −2.87, *p* = .004, 95% CI [−0.20, −0.04]). Although an indirect effect was expected, the direction of the relationship is surprising as higher perfectionistic strivings is often correlated with higher psychological distress in the literature.

The total effect between perfectionistic strivings and flourishing was statistically significant and positive (*b* = 0.16, *SE* = 0.07, *z* = 2.14, *p* = .033, 95% CI [0.01, 0.30]). The direct effect between perfectionistic strivings and flourishing was positive and not statistically significant (*b* = 0.02, *SE* = 0.06, *z* = 0.25, *p* = .800, 95% CI [−0.10, 0.13]). There was a statistically significant positive indirect effect between perfectionistic strivings and flourishing through self-esteem, not supporting H4 (*b* = 0.14, *SD* = 0.05, *z* = 2.91, *p* = .004, 95% CI [0.05, 0.24]). Similarly, an indirect effect was expected, yet the direction of the relationship is somewhat surprising as higher perfectionistic strivings is often correlated with negative mental health outcomes in the literature.

## Discussion

The aim of this study was to investigate how perfectionistic concerns and perfectionistic strivings relate to psychological distress and flourishing through self-esteem, in line with the Two Continua Model of Mental Health (Keyes, 2002). The total effects indicated that participants with higher perfectionistic concerns reported higher psychological distress and lower flourishing, consistent with previous research (e.g., Eley et al., 2020; Flett & Hewitt, 2015; Geranmayepour & Besharat, 2010; Kahn et al., 2022; Stoeber & Corr, 2016; Wimberley & Stasio, 2013). In both cases, this relationship was mediated by self-esteem which supports H1 and H2. As expected (Dunkley et al., 2012; Juwono et al., 2022; Taylor et al., 2016), participants with higher perfectionistic concerns reported lower self-esteem. Consistent with Li et al. (2010) and Parola and Marcionetti (2023), participants with lower self-esteem also reported higher psychological distress and lower flourishing. In line with similar previous research (Awad et al., 2022; Chai et al., 2020; Kempke et al., 2011), the indirect effects specifically indicate that higher perfectionistic concerns was related to higher psychological distress and lower flourishing through self-esteem. This replicates earlier mediation study findings by Rice et al. (1998) and Ashby et al. (2006) that higher perfectionistic concerns relate to lower self-esteem and higher negative mental health outcomes. Overall, these findings suggest that participants with higher perfectionistic concerns were more likely to have lower self-esteem, and in turn, higher psychological distress and lower flourishing.

Despite the well-established relationship between higher perfectionistic concerns and higher psychological distress (Eley et al., 2020; Kahn et al., 2022; Wimberley & Stasio, 2013), perfectionistic concerns were not directly related to psychological distress, yet rather indirectly related to higher psychological distress through self-esteem. This indicates that perfectionistic concerns may not directly predict all psychological outcomes that it is associated with and that it is important to assess mediators of these relationships.

Further, higher perfectionistic concerns were directly related to higher flourishing. This is surprising given that higher perfectionistic concerns are often associated with lower flourishing (Flett & Hewitt, 2015; Stoeber & Corr, 2016). It appears that higher perfectionistic concerns predict lower self-esteem, which predicts lower flourishing. Again, this suggests the importance of examining mediators in the relationship between perfectionistic concerns and psychological outcomes. This finding extends earlier work by Rice et al. (1998) and Ashby et al. (2006), which primarily focused on negative mental health outcomes, by demonstrating a different pattern of direct associations when both psychological distress and flourishing are examined. In line with previous research (e.g., Ashby et al., 2006; Lasota & Kearney, 2017), the results from the current study suggest that self-esteem may play a large role in the relationship between perfectionistic concerns and psychological distress and flourishing.

There was a non-significant total effect between perfectionistic strivings and psychological distress. Consistent with Stoeber and Corr (2016) findings, participants with higher perfectionistic strivings reported higher flourishing. Participants with higher perfectionistic strivings also reported higher self-esteem, in line with similar previous research (Khossousi et al., 2024; Taylor et al., 2016). Consistent with past research, participants with higher self-esteem also reported lower psychological distress (e.g., Li et al., 2010) and higher flourishing (e.g., Parola & Marcionetti, 2023). Interestingly, the direct effects indicate that higher perfectionistic strivings were related to higher psychological distress, consistent with findings from recent systematic reviews and meta-analysis (Callaghan et al., 2023; Lunn et al., 2023), whereas there was no direct effect between perfectionistic strivings and flourishing. Contrary to H3 and H4 yet consistent with some similar previous research (Chai et al., 2020), the indirect effects indicate that higher perfectionistic strivings were related to lower psychological distress and higher flourishing through self-esteem. These indirect effects are in the opposite directions to what was anticipated. Overall, these findings suggest that participants with higher perfectionistic strivings were more likely to have higher self-esteem, and in turn, lower psychological distress and higher flourishing.

Perfectionistic strivings was not directly related to flourishing, yet higher perfectionistic strivings was indirectly related to higher flourishing through self-esteem. On the surface, this is surprising as perfectionistic strivings are often positively related to negative mental health outcomes (Bills et al., 2023; Callaghan et al., 2023; Lunn et al., 2023; Stackpole et al., 2023). However, an alternative explanation may be that perfectionistic strivings tend to correlate with positive mental health outcomes in university student samples (Geranmayepour & Besharat, 2010; Ståhlberg et al., 2019; Stoeber & Corr, 2016) which may be relevant to this study as the current sample is largely university students. These findings also indicate that higher self-esteem may be more strongly related to flourishing than perfectionistic strivings.

All analyses found absent or small direct effects and statistically significant indirect effects through self-esteem. These findings highlight that an individual’s self-esteem may play an important role in the relationship between perfectionistic concerns and perfectionistic strivings and psychological distress and flourishing.

### Implications

From a theoretical perspective, the findings lend support to the Two Continua Model of Mental Health (Keyes, 2002) which proposes that psychological distress and flourishing are related yet separate continuums. Perfectionistic strivings both increased distress directly and increased flourishing indirectly through self-esteem, demonstrating these distinct continuums. This indicates that perfectionistic strivings can be a risk factor for distress and a potential contributor to wellbeing via different psychological processes, such as self-esteem. It also suggests that the commonly observed association between perfectionistic strivings and negative mental health outcomes in the literature may reflect a tendency to prioritise these negative outcomes and overlook positive mental health outcomes which are essential for a more holistic understanding of mental health.

Clinically, the findings indicate that perfectionism may be related to mental health outcomes through other variables, such as self-esteem. Individuals high in perfectionistic concerns may unduly base their self-esteem on achieving their highly set goals which have been shaped by their perfectionistic standards. When these goals are not met, it may lead to lower self-esteem and higher negative mental health outcomes. Future longitudinal research could evaluate this proposition.

It is also plausible that higher self-esteem may be a protective factor in the relationship between perfectionistic concerns and perfectionistic strivings and psychological distress and flourishing (Tiwari et al., 2023). Higher self-esteem may buffer against distress and promote higher flourishing and wellbeing (Parola & Marcionetti, 2023; Trong Dam et al., 2023). In individuals with higher perfectionistic tendencies, their self-esteem may shape their appraisals of situations that then influence their mental health. For example, higher self-esteem may promote more balanced appraisals (e.g., “Although I did not achieve my full goal, I did achieve X, Y, and Z”), leading to lower psychological distress and higher flourishing. In contrast, lower self-esteem may contribute to negative appraisals (e.g., “I did not achieve my full goal so I am a failure”), leading to higher psychological distress and lower flourishing.

Online cognitive-behavioural therapy programmes targeting perfectionism can reduce perfectionism and improve self-esteem (Kothari et al., 2019), suggesting that future longitudinal research could evaluate causality in this relationship and whether such interventions can enhance flourishing among individuals with high perfectionistic concerns and perfectionistic strivings.

### Limitations and future research

The current study’s findings should be interpreted within the context of its limitations. The correlational study design does not allow causal links to be drawn between perfectionism, self-esteem, and mental health outcomes. The cross-sectional nature of the data also prevents any conclusions about the temporal order between, and context surrounding, perfectionism, self-esteem, and mental health outcomes. Future research should use a longitudinal design to explore causality and temporal links in this relationship. Additionally, this study has a primarily female and university student sample which may limit the generalisability of the findings to the wider population. Given that perfectionistic strivings is often positively related to positive mental health outcomes in university student samples, future research should examine the current variables in a more gender-balanced, general community sample.

## Conclusion

This is the first known study to investigate the mediating role of self-esteem in the relationship between perfectionistic concerns and perfectionistic strivings and psychological distress and flourishing. The current findings suggest that self-esteem may be an important variable in the relationship between perfectionism and mental health outcomes. Future research should examine causality in this relationship as well as the extent to which therapeutic interventions can reduce perfectionism and improve self-esteem and flourishing.

## AppendicesAppendix A. Descriptive statistics for each variable

**Table A1.** Descriptive statistics *(N* = 162).

Variable	M	SD	Range
Age	31.57	15.75	18–90
Perfectionistic Concerns	11.70	3.94	4–20
Perfectionistic Strivings	13.69	3.34	6–20
Self-Esteem	27.40	5.08	13–40
Psychological Distress	20.12	12.36	0–61
Flourishing	6.57	1.49	1.31–9.75

*Note*. *M* = Mean; SD = Standard Deviation.

## Appendix B. Bivariate correlations for all variables

**Table B1.** Bivariate correlations: Pearson’s product-moment correlation coefficient (r) for all variables *(N* = 162).

Variable	1	2	3	4	5	6	7
1. Age	1	−.15	−.48**	−.17*	.45**	−.45**	.41**
2. Gender	–	1	.26**	.13	−.15	.31**	−.13
3. Perfectionistic Concerns	–	–	1	.37**	−.65**	.59**	−.40**
4. Perfectionistic Strivings	–	–	–	1	−.08	.24**	−.01
5. Self-Esteem	–	–	–	–	1	−.74**	.73**
6. Psychological Distress	–	–	–	–	–	1	−.62**
7. Flourishing	–	–	–	–	–	–	1

*Note*. **p < .01 (two-tailed) *p < .05 (two-tailed).
